# Angiogenesis in Lymph Nodes Is a Critical Regulator of Immune Response and Lymphoma Growth

**DOI:** 10.3389/fimmu.2020.591741

**Published:** 2020-12-03

**Authors:** Lutz Menzel, Uta E. Höpken, Armin Rehm

**Affiliations:** ^1^ Translational Tumor Immunology, Max Delbrück Center for Molecular Medicine, Berlin, Germany; ^2^ Microenvironmental Regulation in Autoimmunity and Cancer, Max-Delbrück-Center for Molecular Medicine, Berlin, Germany

**Keywords:** lymphoma, B cell malignancy, angiogenesis, lymph node, tumor microenvironment, reactive endothelium, lymphocyte trafficking, high endothelial venule

## Abstract

Tumor-induced remodeling of the microenvironment in lymph nodes (LNs) includes the formation of blood vessels, which goes beyond the regulation of metabolism, and shaping a survival niche for tumor cells. In contrast to solid tumors, which primarily rely on neo-angiogenesis, hematopoietic malignancies usually grow within pre-vascularized autochthonous niches in secondary lymphatic organs or the bone marrow. The mechanisms of vascular remodeling in expanding LNs during infection-induced responses have been studied in more detail; in contrast, insights into the conditions of lymphoma growth and lodging remain enigmatic. Based on previous murine studies and clinical trials in human, we conclude that there is not a universal LN-specific angiogenic program applicable. Instead, signaling pathways that are tightly connected to autochthonous and infiltrating cell types contribute variably to LN vascular expansion. Inflammation related angiogenesis within LNs relies on dendritic cell derived pro-inflammatory cytokines stimulating vascular endothelial growth factor-A (VEGF-A) expression in fibroblastic reticular cells, which in turn triggers vessel growth. In high-grade B cell lymphoma, angiogenesis correlates with poor prognosis. Lymphoma cells immigrate and grow in LNs and provide pro-angiogenic growth factors themselves. In contrast to infectious stimuli that impact on LN vasculature, they do not trigger the typical inflammatory and hypoxia-related stroma-remodeling cascade. Blood vessels in LNs are unique in selective recruitment of lymphocytes via high endothelial venules (HEVs). The dissemination routes of neoplastic lymphocytes are usually disease stage dependent. Early seeding via the blood stream requires the expression of the homeostatic chemokine receptor CCR7 and of L-selectin, both cooperate to facilitate transmigration of tumor and also of protective tumor-reactive lymphocytes via HEV structures. In this view, the HEV route is not only relevant for lymphoma cell homing, but also for a continuous immunosurveillance. We envision that HEV functional and structural alterations during lymphomagenesis are not only key to vascular remodeling, but also impact on tumor cell accessibility when targeted by T cell–mediated immunotherapies.

## Introduction

Lymph nodes (LNs) are strategically positioned hubs of the immune system, connecting the lymphatic system with the blood circulation, filtering antigens and organizing the encounter of lymphocytes with antigen presenting cells (APCs). The LN parenchyma is tightly packed with numerous types of immune cells and susceptible for their immigration and release during conditions of homeostasis, inflammation and tumor transformation. The complex reciprocal interactions of stromal cells and immune cells in LNs shape an adapted microenvironment that supports angiogenesis and increased LN vascularization (1). Although numerous studies reported vascular remodeling and expansion in LNs upon pathogen or tumor cell encounter, the detailed mechanisms and the participating cells of these angiogenic processes are not yet identified. In this review, we delineate the current state of knowledge and propose probable cellular interactions that participate in vascular growth in LNs. In particular, we will focus on the intricate relationship between immune cells and vascular cells as a major pillar of the tumor microenvironment (TME).

B cell non-Hodgkin lymphoma (B-NHL) is a heterogenous group of hematological malignancies that arise from B lymphocytes at various stages of differentiation. Lymphomas grow in the bone marrow and in the secondary lymphatic organs (SLOs), with a predominance of LNs and spleen, but they can also manifest in non-lymphoid tissues (2). The genetic and epigenetic alterations and the intracellular pathway dysregulations responsible for the pathogenesis and progression of lymphomas have been extensively studied and led to tremendous advancements in therapeutic intervention strategies (3). The idea of tumor dependency on cells in the surrounding of a a priori benign environment and on adapted organ properties goes back to Rudolph Virchow in the 19^th^ century (4). The crucial influence of the cellular context in which lymphoma cells arise and lodge attracts growing interest, and the investigation of the TME became an increasingly appreciated field in cancer research (5, 6). The TME constitutes about half of the tumor mass in indolent follicular lymphoma (FL) and marginal zone lymphoma (MCL), whereas the proportion in aggressive diffuse large B cell lymphoma (DLBCL) is generally lower and scarce in Burkitt’s lymphoma (BL) (7). On the extreme, in classical Hodgkin lymphoma (cHL) only about 2%–3% of the cells comprise the malignant Hodgkin-Reed-Sternberg cells (8). Hence, the composition and the dependency of the different B-NHL and cHL on the TME differ substantially between the entities (7). What distinguishes solid tumors and their metastasis most from lymphoma is that within SLOs, transformed B cells encounter a TME infrastructure that genuinely supports survival of benign B cells. These tissues undergo refinement to the needs of the tumor cells induced by a continuous reciprocal crosstalk of tumor, immune and mesenchymal stromal cells (9).

The complex interactions of transformed B cells and the TME lead to extensive changes of the vasculature within the affected organs, which is considered to have a substantial prognostic impact on the patients’ disease outcome (7, 10). The stromal compartment, mainly comprised of blood vessels, lymphatic sinuses and the fibroblastic reticular network is tightly interconnected and regulated. In some respect, it can be considered to represent a joint structural compartment in which its distinct subcompartments grow and remodel in a synchronized manner (11, 12). While the crucial role of lymphatic vessels during lymphoma growth and dissemination is undisputed (13, 14), here we will highlight the influence of blood endothelial cells (BECs) and the blood vasculature, which comprise the main provider of nutrition for proliferating and differentiating immune and tumor cells. In addition, the blood vasculature shapes a major dissemination route for benign immune and transformed cells (15, 16).

## Expansion of Blood Vasculature in LNs During Development, Inflammation, and Cancer

Tumors often recapitulate developmental traits of tissues in which they arise. The stem cell-like phenotype of many tumors is characterized by gene expression signatures that are associated with embryonic stem cell identity and underlines the close transcriptional relationship between neoplastic and developmental tissue (17, 18). Similar to rapidly developing and growing organs, tumors require blood vessels to access oxygen and nutrients. The initiation of blood vessel expansion, referred to as angiogenic switch, occurs at different stages during tumorigenesis, depending on the tumor type and the respective TME. The onset of neo-vascularization and vascular remodeling is a multifactorial processes orchestrated by activating and inhibiting factors whose balance determines whether BECs stay quiescent or get activated (19).

**Figure 1:**
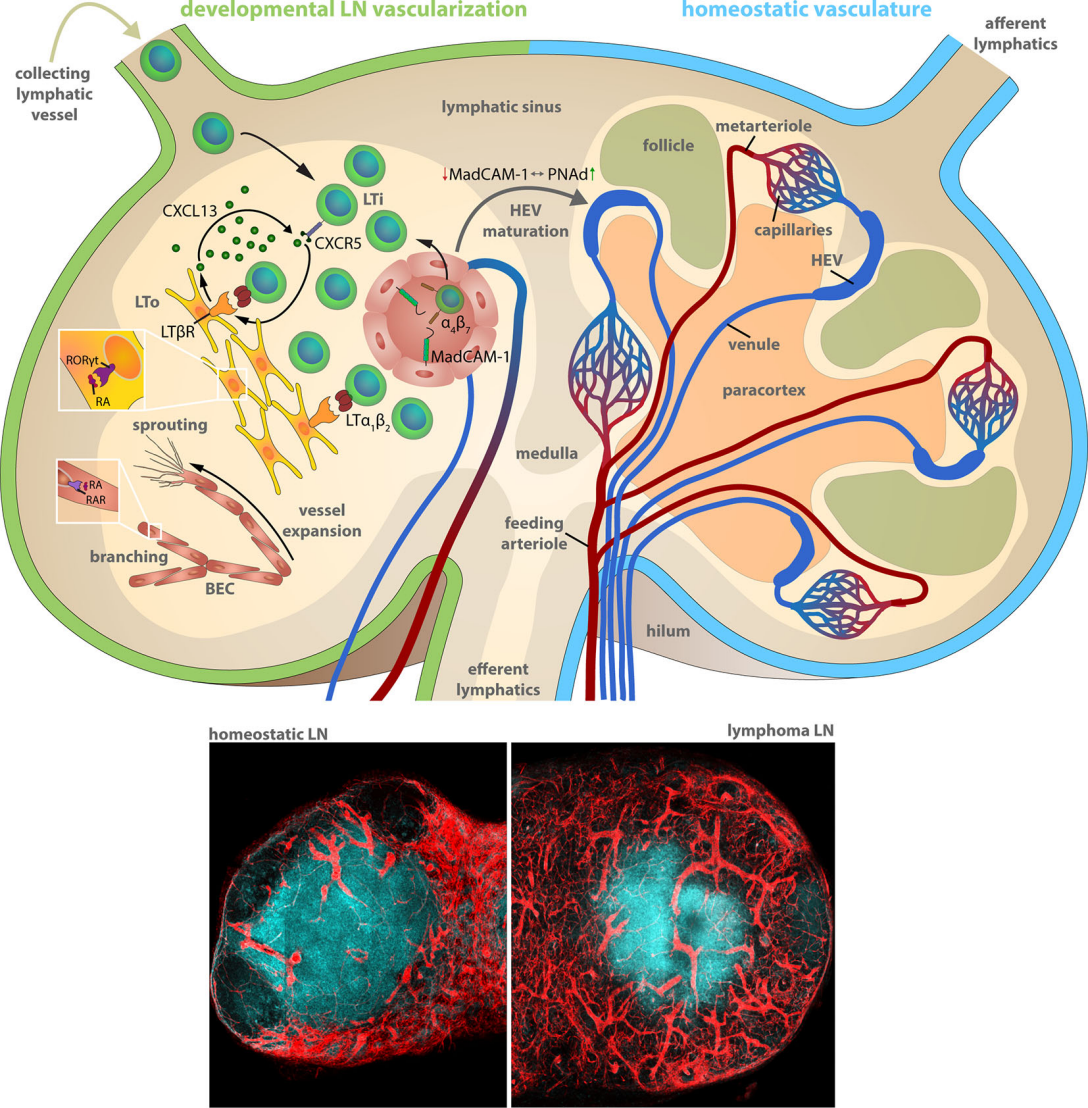
Lymph node vascularization in development and under homeostatic conditions. The LN compartments during LN development (left) and homeostatic conditions (right). Left: Lymphoid organogenesis is driven by recruitment of Lymphoid tissue-inducer (LTi) cells that stimulate lymphoid-organizer (LTo) cells via lymphotoxin (LT) α_1_β_2_ - LT β receptor signaling, which secrete LTi-recruiting CXCL13 in turn. LTi recruitment from the blood circulation and the afferent lymphatics accumulates LTi cells within the LN anlagen resulting in a self-amplifying process of LN development. α_4_β_7_ integrin-expressing LTi recruitment and extravasation utilizes the mucosal vascular addressin cell adhesion molecule-1 (MadCAM-1) on the luminal surface of blood vessels. MadCAM-1 switches to peripheral node addressin (PNAd) expression during differentiation of mature high endothelial venules (HEVs) within peripheral LNs. The formation of the blood vessel network comprises sprouting and branching of expanding blood vessels driven by retinoic acid (RA) stimulation of the RA receptor (RAR) on blood endothelial cells (BECs). Right: The blood circulation enters the LN during homeostatic conditions via the feeding arteriole at the LN hilum, proceeds along the medullary cord and branches into metarterioles that feed the capillary networks around the medulla and at the subcapsular sinus. HEVs are post-capillary venules with a characteristically enlarged vessel diameter. The venous backflow leaves the LN in a bundle of venules at the hilum. Bottom: Representative histochemistry sections (vessels: *Cadherin5* ^fluoresent_reporter^, red) of murine LNs during homeostasis and during progression of a murine high-grade B cell lymphoma.

Therefore, it is useful to recapitulate the essential steps during development to understand the basal mechanisms of the microenvironmental remodeling in LNs. Blood vessels in LNs reside within the stromal scaffold and are crucial for the delivery of oxygen, nutrients, and cells. The critical delivery function was demonstrated by the rapid occurrence of necrosis in LNs upon ablation of the arterial feeding vessel in rats (20). During development (**Figure 1**), LNs evolve from budding lymphatic veins that form a primordial lymph sac, also known as LN anlagen. Studies with transgenic mice lacking lymphatic vessels due to the deficiency for the transcription factor (TF) Prox1 or appropriate lymphangiogenesis factors, e.g., vascular endothelial growth factor-c (Vegfc^+/−^), revealed a compromised LN development (21, 22). LN anlagen recruit hematopoietic lymphoid tissue-inducer (LTi) cells, which in turn stimulate local mesenchymal cell differentiation into lymphoid tissue-organizer (LTo) cells. The accumulation and interaction of lymphotoxin (LT) α_1_β_2_ on LTi cells and LTβ receptor (LTβR) expressing LTo cells results in a self-amplifying loop of LTi recruitment and LTo differentiation that drives the LN development (23). The lymphoid organogenesis is accompanied by the maturation of blood vasculature driven by locally generated retinoic acid (RA) (24). RA is presumably provided by neurons localized adjacent to the developing LN. It directly regulates the proliferation of endothelial cells, but also induces CXCL13 expression in LTo cells via binding to the RA receptor-related orphan receptor (RORγt). CXCL13 in cooperation with its receptor CXCR5 is the exclusive inducer of the initial clustering of LTi cells in LN anlagen independently of LT-LTβR signaling (25). A ubiquitous expression of the mucosal addressin cell adhesion molecule-1 (MadCAM-1) on developing venous blood vessels in the LN mediates the directed immigration of the α4β7 integrin expressing LTi cells (26–28). Notably, the expression of MadCAM-1 in peripheral LNs of newborns switches during the formation and maturation of high endothelial venules (HEVs) into the expression of the peripheral node addressin (PNAd). PNAd expression marks the completion of the maturation of the postcappillary vessels to highly differentiated HEVs that provide all prerequisites for the functional transmigration of blood-borne lymphocytes into the developing and homeostatic LN (**Figure 1**) (26).

The vascular system of LNs in adult mammals is composed of arteries, capillaries, post-capillary venules and veins (29). Arteries are characteristically located at the periphery of the LN. The feeding arteriole enters the LN at the hilum and exhibits a gradual decrease in diameter at its few branching points alongside the medullary cords until it reaches the subcapsular capillary network. Capillaries form a dense network under the subcapsular sinus and around the medullary cords, whereas they are markedly less dense in cortex regions and sparse within the paracortex under homeostatic conditions. The vessel diameter abruptly increases at the capillary to post-capillary transitions. These post-capillary venules, referred to as HEVs, are primarily located within the cortex in the interfollicular space. HEVs form loop-like structures following a centripetal course that ends in transitions to veins at the corticomedullary junctions. Finally, a bundle of larger main veins leave the LN though the hilum (**Figure 1**) (29, 30).

Tumor growth is often accompanied by the ingrowth of blood vessels and the formation of a vascular network, consistent with the need for malignant cells to have access to the circulation system. Tumor vascularization occurs either through co-option of the pre-existing vasculature, or by induction of neovascularization. Vessel co-option is a non-angiogenic process in which tumor cells utilize pre-existing blood vessels of surrounding tissue to support tumor growth, survival and metastasis (31). In contrast, neovascularization involves a series of complex and sequential events: (I) activation of microvascular endothelial cells, (II) enzymatic degradation of the vascular basal membrane, (III) gradual degradation of other extracellular matrix (ECM) components, (IV) endothelial cell migration and proliferation, (V) lumen formation within neo-sprouts, (VI) branching of the neo-vessel, and (VII) formation of a functional vessel network by fusion with neighboring vessels to initiate blood flow (32, 33). Located at the leading edge of the vascular sprout, tip cells form cellular protrusions or filopodia to guide migration toward a source of angiogenic growth factors. Simultaneously, they signal to adjacent endothelial cells via Delta-like ligand (DLL)-Notch interactions not to adapt the tip cell phenotype, but to maintain the proliferative stalk cell phenotype and to form a vascular lumen (34, 35). The vascular endothelial growth factors (VEGFs) are the major contributors to angiogenesis. The local secretion of VEGF-A and its gradient forming deposition on the ECM triggers endothelial tip cell formation via binding to VEGFR2, resulting in endothelial cell proliferation and migration and eventually, formation of tube structures resembling new capillaries (35–38). VEGF-B, VEGF-C, and VEGF-D are other members of the VEGF family of which VEGF-C plays a critical role upon LN remodeling because it is the most potent inducer of lymphangiogenesis as a ligand of VEGFR3. VEGFR3 is known for its involvement in physiological and tumor-associated lymphangiogenesis and lymphatic metastasis (39, 40). Apart from lymphatics, VEGFR3 is highly expressed at the leading-edge of BECs that undergo sprouting (41) and was recently shown to coregulate the expansion of the blood vessel network in LNs in a *Myc*-driven high-grade B cell lymphoma mouse model (42). Fibroblast growth factors (FGFs) stimulate endothelial cell migration and proliferation in a very potent manner, which in *in vitro* experiments even exceeds the stimulation capacity of VEGF-A (43, 44). FGF-1 stimulates proliferation and differentiation of all cell types necessary for the formation of arterial vessels, including endothelial and smooth muscle cells. The angiogenic potency of FGFs extends to prompt fibroblastic cells (e.g., pericytes, smooth muscle cells, and mural cells) and recruits them for vessel formation and maturation during tumorigenesis (45). FGF-2, the second most abundant growth factor of the FGF family, promotes endothelial cell proliferation and the physical organization of the endothelial cell tube-like formation during developmental vessel assembly (46, 47).

The integral investigation of the highly complex vascular network and the unique features of its parts in context of the compartmentalized architecture of the LN has long been a challenge for microscopic image analysis. Because higher order anatomical data sets were obtained from such advanced optical imaging approaches, algorithms for data handling were also demanding to generate. Over the last couple of years, novel tissue preparation methods (48, 49), imaging systems and computational rendering strategies evolved, which enable contextual and organ-wide topological analyses in three-dimensional spaces and over time. In particular, optical projection tomography (OPT) and light sheet microscopy have been established to study anatomical and functional features of LN, e.g., to quantify capillary and HEV structures and their contextual relationship to B cell follicles and dendritic cells (DCs) throughout the organ (50–52). A combination of microscopic imaging and computational modulation of the hydrodynamic properties of vessels in LNs revealed a tight connection of the hydraulic conductivity between lymphatic and blood vessels and the respective hydrodynamic conditions within the LN. These biophysical conditions are vital for inter- and intra-LN transport mechanisms and immunological functions, and most likely for lymphoma B cell dissemination and immunosurveillance as well (53, 54). Up to date, these dynamic conditions are not easy to mimic in organoid models. However, in an early 3D organoid model mimicking a LN exposed to tissue injury or inflammation, the interstitial flow affected the fibroblastic reticular cells (FRCs) that enwrap conduits transporting fluid from the subcapsular sinus to HEVs. Blocking this flow led to CCL21 downregulation, indicating that increased lymph flow as a hydrodynamic factor acts on the paracortex and thus, affects the remodeling and functionality of conduits and FRCs (55). In line, mechanosensing of conduit flow deprivation by FRCs in Peyer’s patches resulted in dysfunctional HEVs and disturbed mucosal immune responses (56). Similar processes are also conceivable during lymphoma growth within LNs, where a gradual loss of HEVs in numerous B-NHL was described many years ago (57). A comprehensive and continuous blood vessel network of LNs under homeostatic conditions has been revealed (54, 58) and brought up an analysis pipeline for detailed and whole-organ investigations of the LN vasculature upon perturbations through inflammation, lymphoma homing and LN solid tumor metastasis. Recently, utilization of single cell transcriptome analysis methods revealed a broad overview of the heterogeneity of ECs throughout several different murine organs, including the spleen and LN as representatives for SLOs (59, 60).

## The Blood Vasculature is Part of The Reactive Stromal Infrastructure During Inflammation and Cancer Development

Inflammation, vessel reorganization and angiogenesis are intimately connected processes. In adults, angiogenesis usually occurs during pathological settings such as infection, wound healing and cancer. Notably, hematopoietic cells and endothelial precursors share common CD34^+^ stem and progenitor cells (61).

Growth of solid tumors is typically associated with inflammation that triggers tissue-protective and pro-tumorigenic mechanisms. Inflammatory responses in normal tissue and cancer are initiated and maintained by local tissue or cancer associated macrophages (TAMs) and DCs. Sustained inflammation further leads to recruitment of bone marrow–derived monocytes, neutrophilic granulocytes, myeloid-derived suppressor cells (MDSC), and tissue or tumor infiltration of lymphocytes from the SLOs. Especially cytokines and chemokines, transcriptionally regulated downstream of NF-κB signaling pathways in immune cells, promote cell survival and proliferation, recruit more immune cells and re-shape the TME. Pro-inflammatory cytokines like IL-6, TNFα and IL-17, increase the proliferation rate of other inflammatory immune cells and prime the tumor to overcome suboptimal microenvironmental conditions including lack of nutrients, growth factors and hypoxia (62). Inflamed tissue and solid tumors are often characterized by insufficient oxygen supply that triggers angiogenesis. Hypoxia, which is the major driver of vascular alterations in solid tumors, stabilizes the TF HIF-1α, the master regulator of pro-angiogenic factor expression such as VEGFs, CXCL12, and COX-2 (63–65). The presence of a constant pro-angiogenic milieu in solid tumors often causes a disturbed maturation and pruning of blood vessels. The division in arterioles, capillaries and venules can be deficient and results in an aberrant distribution of vessel caliber, influencing the blood flow. Morphologically, a poorly organized, malformed vessel network develops under these conditions (66, 67). The endothelial junctions in such malformed networks are often defective and lead to enhanced permeability and elevated interstitial fluid pressure (68). Pericytes can be partially detached and newly build blood vessels often fail to recruit sufficient pericyte coverage, causing an unevenly distributed basement membrane, vessel fragility, and risk of hemorrhage (69, 70). Besides the structural and functional defects, the specific transcriptional response of tumor vasculature is not only related to angiogenesis and vessel integrity, but affects endothelial activation and recruitment of leukocytes as well. Pro-angiogenic signaling leads to endothelial anergy, reduced response to pro-inflammatory signaling and decreased expression of adhesion molecules and chemokines necessary for capture and trans-endothelial migration of leukocytes (71, 72).

In LNs, which are the authochthonous environment for most B-NHL, the pre-existing vasculature takes part in the massive remodeling process during immune responses, best studied for strong inflammatory stimuli in mice (50, 73, 74). LNs are plastic organs able to expand to a multiple of their normal size within days including an extensive remodeling of the vascular-stromal compartment. The rapid expansion of the LN size and cellularity includes early events of remodeling of the feeding artery, causing an increased blood flow and LN hypertension accompanied by an increase of the vascular permeability (75, 76) and increased interstitial pressure. The capillary network within the cortex and medulla expands toward the paracortex, and post-capillary venules are reorganized (30). Skin allograft-draining LNs in rats exhibited a progressive elongation and branching of HEVs resulting from focal proliferation of endothelial cells in the transition zone from high to low endothelium (77). Several years later, Bajénoff and colleagues revisited these observations and investigated the BEC proliferation applying a multicolor fluorescence fate-mapping mouse model. They found similar proliferation foci in post-capillary venules as proposed by Anderson and Anderson. In addition, an extensive expansion of the LN vasculature relying on the sequential assembly of endothelial cell proliferative units upon inflammation was observed. Clonally proliferating HEV cells (73) and capillary resident precursors (60) comprised local progenitors for HEV elongation and capillary neo-vessels during BEC turnover and vessel sprouting. Interestingly, recruitment of bone marrow–derived endothelial cell progenitors did not contribute to the local LN vascular alterations in this model. LN expansion stimulated by several immunization strategies in mouse experiments, e.g., bone marrow–derived DCs (78), ovalbumin/complete Freund’s adjuvant (OVA/CFA) (79), OVA/alum (80), oxazalone (11), and lymphocytic choriomeningitis virus (LCMV) infection (50) indicated similar courses of vessel expansion, starting with early proliferation events that last for up to 5–8 days. The remodeling eventually ends with a gradual re-establishment of the vascular endothelial cell quiescence, a normalization of the vascular bed and restoration of the normal LN size (30, 73, 78).

## Immune Cells are Mediators of Angiogenesis

**Figure 2:**
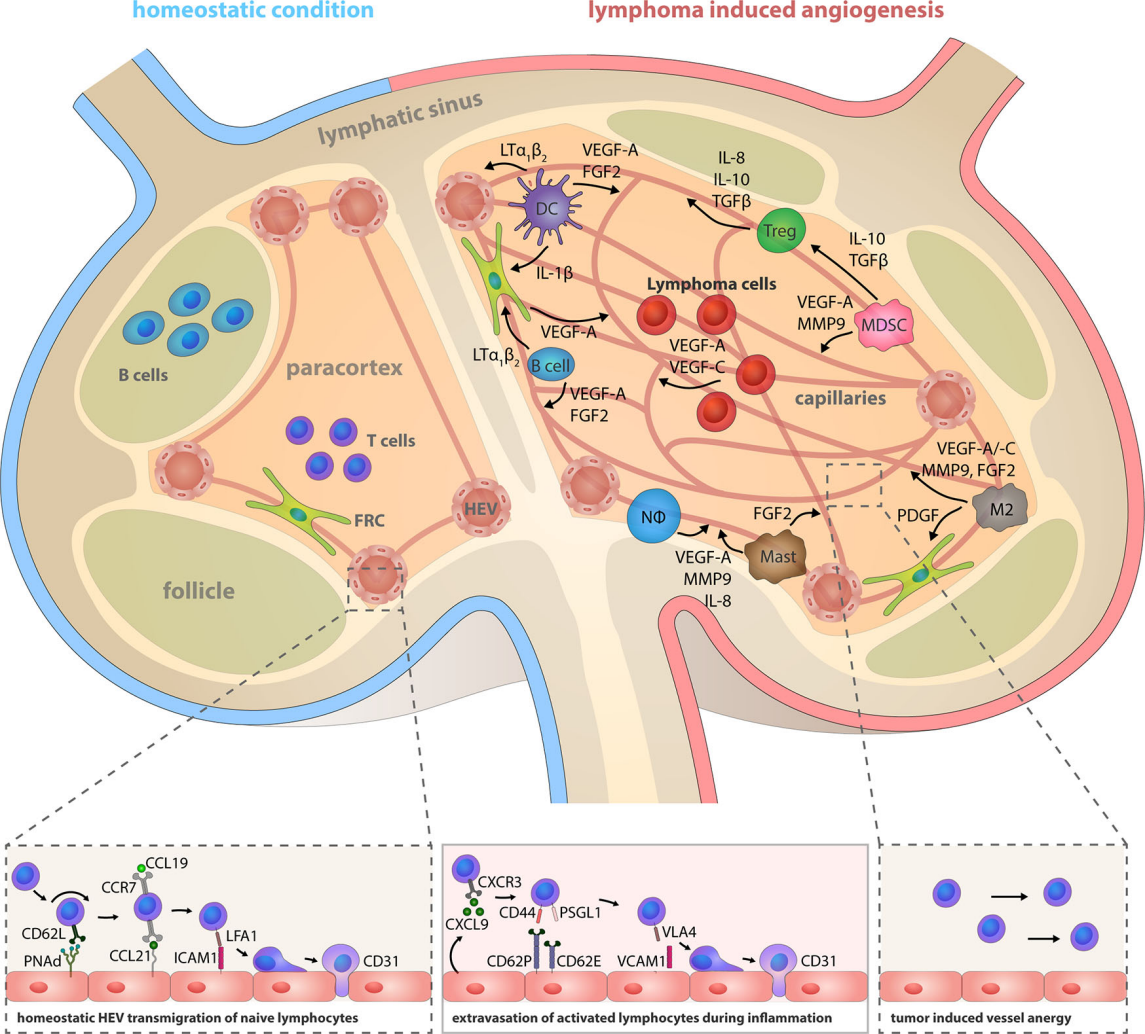
Lymphoma induced angiogenesis in LNs and participating immune cells. Top: The LN compartments represented under homeostatic conditions (left) and lymphoma-activated angiogenesis (right). Lymphoma growth is characterized by a strong LN volume expansion and blood vasculature growth. Remodeling of the stromal infrastructure involves an increase of the microvessel density (MVD), as effectuated by direct angiogenic stimulation through lymphoma B cells cells, but concomitantly also through reciprocal crosstalk of cells in the TME and recruited immune cells. Notably, the initiation of the angiogenic switch in lymphoma is independent from hypoxia-induced HIF1α pathway activation. Tumor polarized DCs (CEBP/β^high^) control the HEV differentiation status via LTα_1_β_2_ and LIGHT presentation; they release IL-1β and hereby take part in the blood vessel growth by inducing VEGF-A expression in FRCs. They also secrete the angiogenic factors VEGF-A and FGF2. B cells express LTα_1_β_2_, which exerts minor effects on HEVs, but a predominating stimulatory effect on FRCs. Expression of the chemokines CCL2, CXCL12, and MIF recruits additional immune cells into the LN. Regulatory T cells (Tregs), myeloid-derived suppressor cells (MDSCs), M2-polarized macrophages, neutrophils and mast cells are capable of producing the pro-angiogenic factors VEGF-A, VEGF-B, VEGF-C, MMP9, IL-8, IL-10, TGFβ, and FGF1/2. Bottom left: HEVs express PNAd, CCL21, and ICAM1 and thereby constitute the transmigration routes for lymphocytes under homeostatic conditions. Interaction of CD62L, CCR7, and LFA-1 on naïve lymphocytes with these HEV-associated surface receptors and chemokines initiates lymphocyte rolling, HEV wall adhesion and eventually, transmigration into the LN parenchyma. Bottom middle: Inflammatory vessels in reactive LNs recruit activated lymphocytes by CXCL9 secretion and replace the homeostatic receptors on endothelial cells with CD62P, CD63E, and VCAM1 that are interaction partners of leukocyte-expressed CD44, PSGL1, and VLA4. Bottom right: The lymphoma induced expansion of the blood vessel network favors the assembly of smaller anergic endothelium that is insufficiently equipped for lymphocyte extravasation.

Both innate and adaptive immune cells have an intricate relationship with angiogenesis. They are involved in regulation of BEC proliferation, migration and activation and they provide a large spectrum of pro-angiogenic mediators apart from their genuine immunological function. Hence, immune cells induce, support or antagonize angiogenic processes during inflammation and tumor growth (**Figure 2** and **Table 1**) (124, 125). Angiogenesis is also important for the progression of B cell lymphoma, however the role of angiogenic factors and the composition of pro-angiogenic immune cells within LNs varies between different entities.

**Table 1:** Immune cells derived pro-angiogenic factors.

Cells	Condition	Angiogenic Factors	Reference
MΦ	Inflammation (mouse, LPS, LTA/MDP)	VEGF-A/C/D	(81)
	Development (zebra fish)	VEGF-A	(82)
	Inflammation (mouse, OVA/CFA)	IL-1β via FRCs	(83)
	Hypoxia (in vitro)	VEGF, bFGF, CXCL8, COX2, HGF, MMP12	(84)
	Mouse/chicken angiogenesis model	MMP2, MMP9	(85)
	Human Monocytes (*in vitro*)	VEGF-A	(86)
	Mouse Matrigel Assay (*in vivo*)	IL-1β	(87)
	Mouse Matrigel Assay (*in vivo*)	FGF2, PlGF	(88)
	Human atherosclerotic plaques	VEGF-A	(89)
	Mouse solid tumors	PDGFβ	(90)
	Squamous carcinoma	VEGF-C	(91)
	human cell lines *in vitro*	TP	(92)
	Ovarian carcinoma	MMP9	(93)
	Breast carcinoma	VEGF-A	(94)
DC	OVA/CFA inflammation (mouse)	VEGF-A	(95, 96)
	Inflammation (mouse, LPS)	PGE_2_	(97)
	LPS, PGE_2_ *in vitro* (mouse)	FGF2	(95)
	development and homeostasis (mouse)	LTα_1_β_2_	(98, 99, 100)
	Inflammation (mouse, OVA/CFA)	IL-1β via FRCs	(83)
	co-culture with NK cells (in vitro)	VEGF-C	(101)
	Il-10 stimulation (in vitro)	Osteopontin	(102)
NΦ	Mouse Matrigel Assay (*in vivo*)	VEGF-A, MMP9	(103)
	Human cells, angiogenesis assay (in vitro)	VEGF-A, IL-8	(104)
	Mouse wound healing assay	VEGF-A	(105)
MC	human skin	VEGF-A, IL-8, MCP-1	(106) (107)
	Human lung mast cells (in vitro)	MMP9, VEGF-A/B/C/D	(108)
	Thyroid cancer	IL-8	(109)
MDSC	Mouse tumor models	VEGF-A, G-CSF, MMP9	(110)
	Mouse melanoma model	VEGF-A	(111)
	Mouse ovarian cancer model	VEGF-A	(112)
	Multiple myeloma mouse model	MMP9	(113)
	Colorectal cancer mouse model	MMP9	(114)
T cells	Inflammation (mouse, OVA/Montanide)	LTα_1_β_2_ via FRCs	(115)
	HUVECs (in vitro)	GM-CSF, IL-8	(116)
	Ischemia mouse model and *in vitro*	IL-10, amphiregulin	(117, 118)
	Type 2 Diabetes	Amphiregulin, IL-10	(119)
	Systemic sclerosis	IL-8, MMP9, VEGF-A induce EPCs diff.	(120, 121)
	Hypoxia (*in vitro*, ovarian cancer)	VEGF-A	(118)
B cells	Inflammation (mouse, LPS)	VEGF-A	(122)
	Inflammation (mouse, OVA/Montanide)	LTα_1_β_2_ via FRCs	(115)
	Virus infection (mouse, LCMV)	LTα_1_β_2_	(50)
	*In vitro* tube formation	VEGF-A, FGF2, PDGFA	(123)

MΦ (macrophages), DC (dendritic cells), NΦ (neutrophils), MC (mast cells), MDSC (myeloid-derived suppressor cells).

A leading immune cell source for growth factors and chemokines to promote angiogenesis under inflammatory and tumorous conditions are myeloid cells (126). **Macrophages** are phagocytic immune cells and important regulators of tissue homeostasis, morphogenesis and repair. In LNs, macrophages are an abundant immune cell population that is divided into subcapsular sinus macrophages (SSM), medullary sinus macrophages (MSMs), and medullary cord macrophages (MCMs) (127). Monocytes from the blood stream and macrophages from LN remote tissues (e.g., bone marrow) infiltrate the LN attracted by a variety of chemotactic factors, among others CCL2, CXCL12, and the macrophage migration inhibitory factor (MIF) (128–130). Tumor-associated macrophages (TAMs) play a prominent role during progression of chronic lymphocytic leukemia (CLL) by supporting tumor cell survival (131) and regulation of the TME (132). The presence and polarization of macrophages during CLL is critical for the tumor progression, as indicated by a CLL-associated skewing of T cells toward antigen-experienced phenotypes and T cell exhaustion, which could be reversed by monocyte and macrophage depletion. Thus, interference with macrophage polarization in CLL turned out to be a promising target for immunotherapy (133). Similar to TAMs in leukemia, macrophages likely support angiogenesis in lymphoma as well, both in cHL and B-NHL. M2-polarized macrophages induce an immunosuppressive milieu in cHL, comprising the majority of the PD-L1 expressing cells, located in close proximity to the Hodgkin Reed Sternberg (HRS) cells (134). In this disease-defining tumor cell population, high frequencies of alterations on chromosome 9p24.1, involving copy number gain and amplifications, have been shown to increase the abundance of the PD-1 ligands, PD-L1 and PD-L2 (135). Furthermore, Epstein-Barr virus (EBV) infection can increase expression of PD-1 ligands in cHL as well (136). The TAM derived PD-L1 in conjunction with the HRS-cell derived PD-1 ligands PD-L1 and PD-L2 may neutralize the anticancer activity of PD-1+ T cells and natural killer cells, a process that can be reversed by utilizing PD-1 blocking antibodies (137). TAMs were also frequently found in FL and DLBCL, among them often polarized and pro-angiogenic M2-like macrophages, which secrete angiogenic factors and re-arrange the ECM by matrix metalloproteinase (MMP) release for vascular expansion (138, 139).

Regarding angiogenesis, macrophages play a crucial role at each step of the angiogenic cascade, starting from blood vessel sprouting to vessel maturation and remodeling of the vascular network. Pro-inflammatory conditions polarize classical activated macrophages (M1), whereas anti-inflammatory conditions give rise to alternatively activated macrophages (M2) including angiogenesis associated macrophages (140–142). For example, in cHL HRS-cell derived TGF-β, IL-13 and M-CSF educate monocytes or TAMs to become immunosuppressive M2-polarized TAMs (Aldinucci D, Casagrande N, 2016, Cancer Letters; Skinnidder and Tak Mak, 2002, Blood).

Macrophage-derived MMP2 and MMP9 proteases cleave the ECM to break open matrix remodeling to pave the way for endothelial sprout migration (85). Pro-angiogenic growth factors, such as VEGF-A, MMPs, IL-1β, FGF2, and transforming growth factor beta 1 (TGFβ1), are part of the repertoire secreted by macrophages in a pro-angiogenic milieu (85–89). Upon hypoxic conditions that lead to HIF1α activation, macrophages are able to establish capillary-like networks in which they line a vessel micro-tunnel and express lineage aberrant endothelial markers such as CD31, von Willebrand factor and Cadherin-5, leading to the assumption that macrophages may transdifferentiate into ECs under specific conditions (143, 144). Macrophages also function as cellular chaperones during anastomosis of vascular sprouts by guiding endothelial tip cells to undergo sprout fusion (82). Such macrophages expressed the markers tyrosine kinase with immunoglobulin-like and EGF-like domains (TIE2) and neuropilin-1 (Nrp1), indicating that they are M2 polarized cells with properties similar to TAMs (82, 145). New blood vessels need to undergo maturation to become functionally stable. A crucial step in this process is the integration of new blood vessels into the established stromal environment and the recruitment of pericytes to strengthen vascular junctions. Macrophages are highly abundant around new blood vessels and help to recruit pericytes by secretion of the platelet-derived growth factor β (PDGFβ) (90, 146).

**Dendritic cells** (DCs) are sentinel cells that connect the innate and adaptive branches of the immune system wherein they have important roles in host defense against pathogens and in generating anti-tumor immune responses. The classical DC compartment of the spleen is comprised of lymphoid tissue-resident DCs, whereas LNs also include non-lymphoid tissue-migratory DCs (147). Especially CD11c^medium^MHCII^high^ DCs are associated with the initiation of vascular expansion after bone marrow–derived DC (BMDC) transfer, whereas CD11c^high^MHCII^medium^ DCs accumulate later in the process and promote the re-establishment of vascular quiescence (78). Apart from their predominant immunologic role as professional APC population, DCs carry a wide range of angiogenic mediators to modulate vascularization. They do so by engaging cognate signaling receptors, such as VEGFR2 on endothelial cells or by recruiting and stimulating adjacent cells or cells of the TME (96). LN-resident DCs are closely associated with FRCs and sample conduit-conveyed antigens within the paracortical and interfollicular zone, where they are located in the proximity of HEVs (148). This spatial proximity suggests that DCs are likely to be a link between immune cells, vasculature and mesenchymal stromal cells. The development, maturation and lineage commitment of DC subsets is differentially regulated by acomplex TF network, depending on homeostatic, inflammatory, and tumorous conditions. We recently demonstrated that the TF C/EBPβ plays a crucial role in murine DC maturation and immunogenic functionality under homeostatic and lymphoma-transformed conditions (149). In the presence of lymphoma cells, enhanced expression of C/EBPβ in DCs was observed which transformed them into an immature, tolerogenic and pro-tumorigenic subtype (150). Such aberrant maturation stages may potentially affect the angiogenic capacities of the DCs as well. The crucial role of DCs for the LN vasculature was elaborated in several studies (12, 78, 79, 96) and revealed the DC-coordinated remodeling mechanisms of blood- and lymph-vasculature, and the FRC network as well. The DC associated increase of VEGF-A in reactive LNs further includes stimulation of a pro-angiogenic program in FRCs and the recruitment of blood-borne cells that participate in the angiogenic process. IL-1β expression by recruited CCR7^+^ CD11c^+^ DCs is associated with the enrichment of VEGF-A expressing FRCs at the border of the LN paracortex (83). The angiogenic role of DCs in lymphoma LNs has not been investigated yet; however, in reactive LNs, resident classic DCs produce biologically active VEGF-A downstream of the inflammation-associated TFs HIF-1α, STAT3, and CREB. HIF-1α and STAT3 are generally related to hypoxic conditions, whereas CREB phosphorylation is the consequence of autocrine and paracrine prostaglandin E2 (PGE_2_) signaling (151). The PGE_2_ production is directly connected to pathogen induced toll like receptor-4 (TLR4) signaling and therefore, delineates the connection of infection induced LN reactivity with angiogenic vessel formation (152). DCs also release other classical angiogenic growth factors like FGF2, endothelin-1 (ET-1), CXCL12, and COX-2. FGF2 activates endothelial cells and induces VEGF-A expression in mesenchymal cells, but also recruits and activates macrophages and mast cells that in turn exhibit angiogenic properties (152, 153). DCs further have the capacity to modulate angiogenesis in an indirect manner through secretion of the monocyte and granulocyte attracting chemokines CXCL8, CXCL1, CXCL2, CXCL3, and CXCL5 (154). The recruited myeloid cells can be triggered to secrete the pro-angiogenic IL-1β by a signaling pathway that includes classical DC-derived osteopontin (155). DCs are not only associated with vascular expansion, but also with the re-establishment of vascular quiescence and stability in the process of reinstallation of the LN homeostasis (78).

**Neutrophilic granulocytes** are the most abundant type of leukocytes throughout the body, representing the pioneering cells that are recruited to injuries and thus, they are frontline defenders against pathogens. Neutrophils infiltrate LNs guided by inflammatory cytokines like IL-1β and TNFα, the complement factors C3a and C5a, along the CXCR4-CXCL12 axis, and eventually they are also attracted by a plethora of inflammatory chemokines (156, 157) (Capucetti, Albano, Bonecchi, Frontiers in Immunology, 2020). Neutrophils are a source of soluble mediators that exert important angiogenic functions. VEGF-A, IL-8, hepatocyte growth factor (HGF), granulocyte colony-stimulating factor (G-CSF), and MMP9 are the most important activators of angiogenesis produced by these cells (103, 158). Interestingly, neutrophils are able to release VEGF-A-enriched granules upon TNFα stimulation and thus, promote vessel growth during inflammation (105). This can become an self-amplifying process since neutrophil-derived VEGF stimulates neutrophil migration via an autocrine amplification mechanism, a process that likely contributes to pathological angiogenesis during inflammation and cancer (159). Human polymorphonuclear granulocytes have been demonstrated to directly induce the sprouting of capillary-like structures in an in vitro angiogenesis assay, mediated by secretion of both pre-formed VEGF from cell stores and de novo synthesized IL-8 (104). In the murine Eμ-*Tcl1* model, mimicking CLL, a tumor-associated neutrophil (TAN) population with a B cell helper-like polarization was identified. Selective depletion of these TANs retarded leukemia progression in SLOs substantially (160).

**Mast cells** (MC) are hematopoietic tissue resident immune cells that are classically recognized as the main effector cell type of IgE-mediated immediate allergic reactions, however they are also frequently associated with tumorigenesis (161–163). According to their protease expression, mast cells are divided in two phenotypical populations: the trypase^+^ chymase^−^ (MC_T_) and the trypase^+^ and chymase^+^ (MC_TC_) cells (164, 165). MC produce several proangiogenic factors, among them VEGF-A, VEGF-B, MMP9, and FGF-2. In addition, mast cells chemotactically respond to VEGF-A and FGF2, indicating that a connection between mast cell accumulation at tumor sites, angiogenesis and tumor growth exists (166, 167).

**Myeloid derived suppressor cells** (MDSCs) contribute to the induction of an immune suppressive and tumor permissive microenvironment. They are frequently found in SLOs like spleen, but they are rare in LNs (168). However, they are able to modulate the L-selectin expression of naïve T and B cells, preventing efficient HEV adhesion, transmigration, and subsequent antigen encounter within LN parenchyma (169, 170). MDSCs promote the formation of T regulatory cells (Tregs), the secretion of immunosuppressive IL-10 and TGF-β, and inhibit the activity of cytotoxic CD8 T cells via expression of arginase-1 (Arg1) and inducible nitric oxidase (iNOS) within the TME of several tumor entities including B cell lymphoma (171, 172). Moreover, MDSCs directly influence the tumor stroma by inducing differentiation of cancer-associated fibroblasts (CAFs) (173, 174). Pro-angiogenic properties of MDSCs during tumor progression have been reviewed recently (175). MDSCs and their progenitors, immature myeloid cells are usually not present in LNs during steady state conditions. However, inflammatory conditions and tumor-derived factors (e.g., CXCL12, GM-CSF, and CCL2) induce the activation and accumulation of MDSC in SLOs (176–178). MDSCs exhibit numerous immunomodulatory properties that have considerable potential to influence angiogenic processes in LNs, either through direct triggering of ECs, or by stimulating leukocyte and stromal cells to establish an angiogenic milieu (175). MDSCs are able to promote tumor angiogenesis through releasing VEGF-A and MMP9. Mouse models suggest that MDSCs integrate into the line of vessel-decorating endothelial cells (179). In mouse melanoma, MDSC contribute to A2B adenosine receptor-induced VEGF-A production (111, 180). VEGF-A in turn stimulates MDSC recruitment from the bone marrow, creating a self-enhancing feedback loop that promotes immunosuppression and vessel growth (112). One of the reasons why several angiogenic tumors occur to be insensitive to VEGF-A-targeted therapy is the presence and recruitment of MDSCs. These cells secrete high amounts of VEGF-A which might lead to neutralization of the VEGF-inhibition and additionally, they establish pro-angiogenic signaling pathways involving several other cells of the TME (110, 181). Moreover, MDSCs limit T cell adhesion and extravasation by VEGF-A stimulated suppression of endothelial ICAM-1 and VCAM-1 expression during tumor angiogenesis (71, 182).

**Lymphocytes**, the major regulatory and executive cell subset of the adaptive immune response are also able to influence angiogenesis during inflammation and cancer, although their specific implications are still enigmatic.

**T cells** comprise different subsets involved in lymphomagenesis, including naive T cells, memory T cells, and Treg cells (183). Several negative regulators of T cell activation act as checkpoints to fine-tune the immune response and regulate hyperactivation. Cytotoxic T lymphocyte antigen 4 (CTLA-4) and programmed cell death 1 (PD-1) are the most potent examples of T cell immune checkpoint molecules (ICB) (184). Cancer patients often display dysfunctional antitumor T cell responses because of the signaling pathways downstream of these receptors. PD-1 and CTLA-4 inhibition are subject of extended clinical studies and led already to impressive response rates in some tumor entities, among them melanoma, non–small cell lung cancer and for hematopoietic tumors, (184–186), in cHL as well (187, 188). By targeting abnormal formation of tumor vessels, anti-angiogenic agents potentially result in an enhanced infiltration of anti-tumor effector cells, making the combination of immune checkpoint inhibitors and anti-angiogenic agents a promising and complementary approach in cancer adoptive T cell therapy (189). On the other hand, as a result of IFNγ and IL-12 stimulation, microvascular endothelial cells express checkpoint molecules like PD-L1 (190, 191). In line, arterial vessels express PD-L1 and PD-L2 after toll like receptor (TLR)-3 activation upon bacterial infection (192). The regulatory and angiogenic effects of CD4^+^ T helper cells (Th cells) are strictly associated with their differentiation. Cytotoxic CD8^+^ T cells and CD4^+^ Th1 cells produce IFNγ that restrains endothelial cell proliferation and induces expression of the angiostatic chemokines CXCL9/10/11 in TAMs (126, 193). *In vitro* studies revealed that Th2- and Th17 cell-conditioned medium triggered endothelial sprouting, whereas medium of Th1 cultures induced vascular regression. Conditioned medium from Tregs had a minor or no effect (116). In vivo, CD4^+^ T cells display opposing effects on vascularization depending on their subset differentiation. Th1 cell-derived IFNγ impairs angiogenesis in ischemic tissue, an effect that is counteracted by regulatory CD4^+^ T cells (Tregs) that antagonize the immunologic Th1 cell response by secreting anti-inflammatory IL-10 and TGFβ. Thus, Tregs display rather indirect pro-angiogenic properties, most likely by paracrine effects on other potentially pro-angiogenic immune cells (e.g., macrophages, DCs, mast cells) (119, 194). T cell recruitment, survival and functionality are highly dependent on tumor-polarized myeloid cells and tumor-derived factors. The typical immuno-suppressive milieu of the TME is characterized by polarizing factors, shifting CD4+ T cell differentiation toward CD4^+^CD25^+^FOXP3^+^ Tregs. In the aggressive Myc-driven murine lymphoma model, this polarization process is promoted by DCs expressing increased amounts of the TF C/EBPβ (144).

In ovarian cancer, Tregs were selectively recruited into the tumor tissue via CCL22 and CCL28 production by the tumor cells and subsequently, Treg-induced secretion of high amounts of VEGF-A to promote endothelial cell proliferation (118, 195). A striking example for Treg recruitment represents cHL; here, Tregs are attracted via the Hodgkin-Reed-Sternberg cell-secreted chemokines CCL17 and CCL22, which engage the Treg-expressed chemokine receptor CCR4 (196), or by the chemokine CCL20 that binds to CCR6 (Baumforth, Birgersdotter, Machado, Am J Pathol, 2008). Th cells and cytotoxic T cells are required to mediate the anti-angiogenic effect of IL-12. IL-12–activated lymphocytes effectuate inhibition of tumor growth and function as anti-vascular agents that release higher amounts of IFNγ while they down-regulate VEGF in neighboring cells (197, 198). Noteworthy, the presence of IFNγ comes at the expense of an induction of PD-L1 on numerous stromal cell types, among them endothelial cells (199, 200); this process is likely to counteract the beneficial effects of IFNγ-secreting effector T cells which may be rendered dysfunctional (201). The infiltration of tumor sites by cytotoxic CD8^+^ T cells is usually correlated with a favorable clinical prognosis, however immunosuppressive conditions can polarize these cells to CD8^+^FOXP3^+^ regulatory cells with similar immunomodulatory and angiogenic properties as CD4^+^ Tregs (202–205). Studies of coronary artery disease and systemic sclerosis found T cells with angiogenic potential in blood samples of patients and demonstrated that these CD3^+^CD31^+^CXCR4^+^ cells (referred to as angiogenic T cells) play a vital role for the colony formation and differentiation of endothelial progenitor cells (EPCs) in the bone marrow (110, 206). Such EPCs have been detected in the circulation and in LN samples from patients with B-NHL as well, although their influence on lymphoma-induced vessel growth is still elusive (207, 208). However, inflammation models argue against a significant functional role of BM-recruited EPCs in LN vascularization (73).

**B cells** are frequently found to be part of the TME (209); however, their role in tumor progression and vascularization is still unclear. They can directly promote angiogenesis by secreting pro-angiogenic factors such as VEGF-A, FGF2, and MMP9 (210), or indirectly by polarizing macrophages to the M2 pro-angiogenic phenotype (211). Transgenic mice (CD19^Cre^/hVEGF-A^fl^) overexpressing human VEGF-A in murine B cells exhibited a VEGF-A induced lymphangiogenesis and an expansion of HEVs in LNs. The authors of the study speculated that the unphysiologically high levels of human VEGF-A might not directly influence the LN lymph- and blood vasculature, but may rather cause an accumulation of pro-angiogenic macrophages (122). In a mouse model of LCMV infection, B cells were shown to be required for LN tissue remodeling and vessel expansion. Surprisingly, the latter was independent of VEGF-A signaling pathways, but required LTα_1_β_2_-expressing B cells (50). A recent study emphasized the angiogenic capacity of a B cell subset during eosinophilic esophagitis and in patients with melanoma. These pro-angiogenic B cells were identified by the surface markers IgG4^+^CD49b^+^CD73^+^ and shown to promote vascular tube formation *in vitro* through VEGF-A, FGF2, and PDGFA expression (123). Taken together, although B cells express VEGF-A and LTα_1_β_2_ during certain conditions, their role in LN angiogenesis is not well understood. Potentially, B cells may exert pro-angiogenic effects themselves, but also through stimulation of other stromal cell types, such as FRCs and macrophages (**Figure 2**).

## B Cell Lymphoma-Induced Vascular Changes are Dependent on The Entity and State of Lymphoma Progression

The clinical importance of angiogenic processes and mechanisms for the growth of solid tumors is well recognized (212, 213). Therapeutic concepts from solid tumors targeting the VEGF-A/VEGFR1/2 axis have been adopted for combinatorial therapies of B-NHL, resulting in rather disappointing clinical outcomes (214, 215). We recently showed that angiogenic processes in LNs in a mouse model of high-grade B cell lymphoma are induced by signaling pathways distinct from solid tumors. In sharp contrast to most solid tumors, lymphoma growth in LNs was not associated with hypoxic conditions or inflammation. Instead, lymphoma affected vessel expansion via the VEGF-C/VEGFR3 and LTα_1_β_2_/LTβR signaling axes (42). In patients, the growth of tumor cells in low-grade B-NHLs is usually exponential for a few months and remains in a steady state as indolently growing tumor mass for years. This indolent lymphoma is considered to be avascular with dormant endothelial cells within the TME. In contrast, high-grade B-NHL progression is often accompanied by a so called “vascular phase”, which represents extensive vascularization of LNs (216, 217). Such intermediate- and high-grade B-NHLs grow exponentially without intermission phase until they reach a mass critical for a patient’s survival. As a clinical indicator of the vascularization, B-NHLs are usually quantified by terms of the microvessel density (MVD). Immunohistology using anti-CD31 antibody staining is still considered the “gold standard” of blood vessel detection, even though there is substantial variation between different studies due to the heterogeneity of the lymphoma stroma and different scoring methodologies. In some cases, the marker CD34 is used to detect the blood vasculature. Notably, lymphatic vasculature also expresses CD31, but at much lower levels (42, 218, 219).

Non-invasive assessment of tumor vascularization *in vivo* is possible by using Doppler sonography, contrast-enhanced dynamic magnetic resonance imaging (dMRI) and positron emission tomography-computer tomography (PET-CT). These techniques do not allow a direct quantification of the blood vessel density but provide information on the functional status of the blood vessels, e.g., vessel integrity, permeability, perfusion and metabolism (220). Another diagnostic approach to detect ongoing angiogenesis *in vivo* is the serological quantification of growth factors. VEGF-A levels in the serum of patients with progressive NHL were significantly elevated in comparison to patients in complete remission (221, 222). Elevated VEGF-A levels have been found in aggressive B cell lymphoma subtypes including MCL, DLBCL, but also in indolent lymphoma, such as CLL and small lymphocytic lymphoma (SLL), respectively (223–225). A variety of commonly used B-NHL cell lines secrete measurable VEGF amounts under serum starvation conditions, whereas other angiogenic factors like the placental growth factor (PlGF) and FGF-2 are not expressed (226). However, the detection of angiogenic factors in clinical serum samples gives no information on the cellular source of these molecules and is not a reliable indicator of angiogenesis in the compartment of interest. Previously, a group of angiogenesis experts published consensus guidelines for the use and interpretation of angiogenesis assays, which involve in vivo, ex vivo explantation, and in vitro bioassays. They explicitly highlighted critical aspects that are relevant for the execution of angiogenesis detection and proper interpretation (227).

**Mantle cell lymphoma** (MCL) is an aggressive B cell neoplasm that comprises 6% of all NHL cases (228, 229). It is susceptible to paracrine signaling from the microenvironment and in turn shapes the microenvironment by secreting soluble factors (230). MCL is genetically characterized by overexpression of the *CCND1*gene, encoding for cyclin D1 (231). Recent studies identiﬁed a subgroup of MCL that has a more indolent behavior with a clinical presentation as leukemic disease, exhibiting minimal LN distribution and a frequent splenomegaly. These tumors also overexpress cyclin D1 but lack expression of the sex determining region-Y-box11 (SOX11), a TF specifically expressed in conventional MCL and associated with an aggressive and angiogenic phenotype (232). These results have been confirmed in MCL patient samples by using immunohistochemistry, demonstrating a correlation between an increased MVD and high levels of SOX11 expression (233). Experiments with MCL tumor xenotransplants in mice, in cell lines, and in primary MCL samples revealed that SOX11 actively modulates angiogenesis by up-regulation of the platelet-derived growth factor α (PDGFα), which is a competent inducer of an FRC-associated pro-angiogenic program (234, 235). Moreover, SOX11 overexpression promotes B cell receptor signaling represses Bcl6 transcription and upregulates PAX5 to avoid B cell differentiation into memory B cells or plasma cells. PAX5 supports tumor cell homing and invasion via up-regulation of CXCR4 and the focal adhesion kinase (FAK) (236–238). The absolute monocyte count in MCL correlated with the prognosis and supports the hypothesis that the TME is relevant for MCL tumor progression (239). CD68^+^ and CD163^+^ macrophages were found in MCL LNs without exception. Substantial numbers of VEGF-C expressing macrophages were found in a mouse xenotransplantation model as well (240). Treatment with the immunomodulator lenalidomide depleted monocytes and VEGF-C expressing macrophages, resulted in impaired functional lymphangiogenesis. However, a relevant impact on lymphoma-associated blood vessel growth in MCL was not investigated in this study. Of note, in human MCL anti-inflammatory and pro-angiogenic CD163^+^ cells (M2-like) outnumbered the more inflammatory CD68^+^CD163^-^ macrophages (233), indicating a propensity to stimulate angiogenesis. This M2-like polarization of macrophages is actively driven by MCL derived CSF-1 and IL-10 (241). MCL cells exhibit increased expression of the T cell, B cell, and monocyte recruiting chemokines CCL4 and CCL5 compared to normal B cells (242). T cell infiltration has been considered as a prognostic marker in MCL in which CD8^+^, and particularly CD4^+^ T cell frequencies are higher in indolent MCL and decrease with more aggressive histological and clinical presentation (243). In contrast, a recent study reported an expanded vascularization of MCL associated with a high infiltration of CD4^+^ and CD8^+^ T lymphocytes (233). The differences might be explained by a weak comparability of data that were either correlated with the clinical outcome, or with the SOX11 expression level in MCL, two hallmarks that are not always correlated. A more detailed T cell characterization of CD4/CD8 T cell subsets is required for a more reliable assessment of the T cell–related influence on angiogenesis and the clinical outcome in MCL. Interestingly, MCL cells itself express the VEGFR-1, providing a strong rationale to target VEGF in order to interfere with angiogenic processes and concomitantly, with autocrine survival signals (230, 244).

Angiogenesis is likely a part of MCL progression, driven by MCL derived PDGFα. Therapeutical interference with PDGFR-β signaling, the receptor for PDGFα, can be achieved with receptor tyrosine kinase (RTK) inhibitors. Some PDGFR-β targeted drugs have been tested in clinical trials for B-NHL but failed to bring significant benefit (217). In contrast, immunomodulating drugs (IMiD) like thalidomide and lenalidomide have anti-angiogenic properties and showed great potential in combination with rituximab for the treatment of untreated or relapsed MCL patients (245, 246).

**Follicular lymphoma** (FL) is the second most common B-NHL, accounting for 20% of all B-NHL cases (247). The disease affects LNs, spleen and frequently also the bone marrow. Neoplastic follicles in FL have a lower proliferative index in comparison to reactive germinal centers. However, the proliferative capacity of FL cells increases gradually with the FL grade. FL progression requires the supporting infrastructure of the follicular TME to maintain survival, a requirement that gets progressively lost in the process of transformation to aggressive DLBCL (248, 249). Follicular dendritic cells (FDCs) are one branch of this supporting infrastructure. They are of mesenchymal origin and represent a crucial stromal cell population supporting the germinal center reaction and maintenance of the B cell follicle in LN and spleen (250, 251). FDC secreted B cell survival factors such as Indian hedgehog (HH), the B cell activating factor (BAFF), and IL-15 are potentially pro-tumorigenic (252, 253). CXCR5-controlled access to FDCs conferred survival and proliferation stimuli to CLL B cells in the murine Eμ-*Tcl1* model, which mimics some aspects of indolent tumor growth (253). Similar to reactive LN follicles, neoplastic follicles in FL preserve the organized FDC network structure at least in early stages of the disease progression (254). FL-FDC cross-talk induces a pro-angiogenic expression pattern in FL cells, including secretion of VEGF-A and VEGF-C (255). This cross-talk is crucially dependent on the phosphoinositide-3-kinase δ (PI3Kδ), providing therapeutic intervention options with PI3K specific inhibitors like idelalisib, which is approved for the treatment of FL, CLL, and SLL (256). The second branch of the supportive infrastructure in follicles are the CD4^+^CXCR5^+^PD1^+^ T follicular helper (Tfh) cells, which provide vital survival signals for FL cells by secreting IL2, IL4, IFNγ, and by CD40L presentation (9, 257). FL cells are further dependent on proliferation and survival signals of the B cell receptor (BCR) in interaction with FDCs and TAMs (258). Elevated numbers of M2-like TAMs are found in the immediate microenvironment of FL cells and neo-vascular sprouts within the follicle (138). However, the prognostic value of CD163^+^ TAMs remains controversial and is highly dependent on the prior course of treatment (259). In sum, FL appeared to be less prone to induce relevant vascular changes, whereas LNs of high-grade B-NHLs exhibited a dense and aberrantly distributed vasculature within the paracortical zone. In contrast to most other B-NHL malignancies in which high levels of pro-angiogenic factors and an increased MVD is associated with an adverse prognosis, high level FL vascularization correlates with a beneficial disease course (260–262). The improved clinical outcome apparently correlated with the increased vascularization, but was surprisingly independent of follicular VEGF-A expression (223, 263). Some studies stated a minor vascular remodeling in FL compared to reactive LN or follicular hyperplasia, or even vascular regression constraining the growth of reactive and neoplastic follicles (260, 264). Therefore, the clinical significance of angiogenesis in FL remains uncertain. In one clinical trial, addition of the anti-VEGF bevacizumab during rituximab treatment of relapsed FL significantly improved the progression-free survival (265). The potential of angiogenesis inhibition upon treatment of FL requires further evaluation in larger clinical trials.

Collectively, according to the data currently available it seems that angiogenesis is important for high-grade lymphoma, but has less impact on indolent FL growth.

**Diffuse large B-cell lymphoma** (DLBCL) is the most common type of lymphoid tumors worldwide accounting for 30% of all diagnosed NHL and characterized by the large size of neoplastic B cells and usually a very aggressive clinical presentation (266). Lenz et al. identified gene expression profiles in LN from patients pre-treated with the combination therapy anti-CD20 antibody, cyclophosphamide, doxorubicin, vincristin, prednisolone (R-CHOP), dividing DLBCL in two distinct subgroups that are predictive of the clinical outcome (267). The “stromal-1” signature includes expression of extracellular matrix (ECM) elements, ECM remodeling factors (*MMP2*, *MMP9*, *M1*-*MMP*, *PLAU*, *TIMP2*) and is associated with a favorable prognosis. The “stromal-2” signature” was found in tumors with an increased MVD and is characterized by markers of endothelial cells (*Pecam1*, *Vwf*, *Kdr*, *Tek*). The latter signature is associated with a poor clinical outcome, emphasizing the critical impact of angiogenic processes on aggressive B-NHL progression. Several studies investigated the clinical consequences of the “stromal-2” signature and confirmed the correlation of a high MVD with an adverse outcome and a shorter overall survival rate (268–271). The relationship between MVD and DLBCL behavior was the object of many studies and was found to be associated with poor prognostic parameters such as splenic involvement, high mitotic rate, and capsular invasion (268–272). Gomez-Gelvez et al. reported contradictory results, showing that high MVD is associated with rather better progression-free survival (PFS) and event-free survival (EFS) (273). Several other studies also failed to draw a connection between the MVD, tumor grade and prognostic outcome (274–277). A DLBCL mouse xenotransplantation model demonstrated that the inhibition of the paracrine VEGFR-2 pathway reduced growth of an established lymphoma and correlated with decreased tumor angiogenesis (226). DLBCL cells often overexpress the phosphodiesterase 4B (PDE4B), which intracellularly catalyzes the hydrolysis of cyclic-AMP (cAMP). The cAMP-PDE4B axis modulates signaling of PI3K and AKT and therefore acts upstream of VEGF-A expression. Experiments with genetically or pharmacologically inhibited PDE4B resulted in decreased VEGF-A expression in lymphoma cells and reduced angiogenesis in the Eµ-*Myc* high-grade lymphoma mouse model (278).

In a gene expression study on relapsed or refractory DLBCL, patients with the ABC-like DLBCL subtype that had low VEGF_121_ isoform expression, exhibited a significantly better overal survival than those with high VEGF_121_ gene expression levels (279). Interestingly, VEGF_121_ low transcript levels were associated to a gene signature reflecting immune response and T cell activation.

DCs are likely a major source of VEGF-A in LNs with DLBCL. Functionally, DCs could be involved in lymphoma TME remodeling, but their number in DLBCL LNs is significantly lower than in reactive LNs. Lower expression levels of the LN homing receptors CD62L and CCR7 in DCs in LNs of DLBCL patients were thought to result in reduced DC immigration. However, it remains elusive if the DCs lose the receptor expression upon arrival in the LN, or whether these cells are recruited via alternative routes (280). In an aggressive *Myc*-driven lymphoma model in mice a tumor-specific DC differentiation occurs that promotes tumor cell survival and favors the maturation of monocytic-derived DCs (MHCII^medium^) (149, 150). Alongside tumor repressing M1 macrophages, “alternatively” activated M2 macrophages exhibit angiogenic capacities, they are frequently found in DLBCL and often correlate with a poor prognosis (281–283). Although numerous studies reported an association between TAMs and MVD in DLBCL, others could not find a correlation between CD68^+^ macrophages and an increased MVD (270). Such controversies can probably be best explained by variabilities in the methodological approaches. The macrophage marker CD68 represents M1 and M2 macrophages and therefore, produces inaccuracies in the interpretation of studies concerning the macrophage-MVD correlation. The addition of the marker CD163, which rather recognizes M2 activated macrophages, including angiogenic macrophages, provides a more reliable view on the role of macrophages in DLBCL (284). Elevated numbers of macrophages have been correlated with poor prognosis in DLBCL (282). However, in therapeutic setting macrophages are required to confer treatment effects when patients were treated with anti-CD20 antibody (e.g., Rituximab). Here, macrophages mediate tumor cell depletion via the macrophage Fc-gamma receptor (FcγR) expression (215). Another abundant immune cell population in LNs of DLBCL patients is mast cells with a predominance of MC_T_-type (tryptase-positive) cells. MC_TC_-type (tryptase-positive and chymase‐positive) and CD4^+^ Th_2_ were shown to express IL-4 in DLBCL and therefore, they may actively promote survival of the tumor cells (285). Hedström and coworkers examined 154 DLBCL cases and suggested that the infiltration of mast cells reflects the inflammatory immune response of the endogenous anti-tumor defense and is therefore related to a favorable outcome (286). The gradual increase of the MVD was correlated with an increasing number of mast cells. Although mast cells are considered to be bystanders in tumor immunology, additional pro-angiogenic effects of these cells are likely as they secrete relevant amounts of different VEGFs, FGF-2, trypase, and granzyme B. The latter has a pro-angiogenic effect via the enzymatic mobilization of ECM-bound FGF-1 (287, 288). The wide range of physiological conditions and tumor entities that include mast cell-supported angiogenesis and the respective recruitment and signaling pathways were excellently reviewed by Ribatti et al. (289).

Apart from the direct effect on immune and tumor cells, surprisingly, the application of the VEGF-A inhibiting antibody bevacizumab to R-CHOP therapy increased adverse cardiac events, yet without increasing the therapeutic efficacy in DLBCL patients (214, 215). From these studies it can be inferred that the increased MVD in DLBCL patients may be simply a correlation with minor importance for the disease course, or that other non-VEGF-A angiogenic pathways prevail and cause enhanced vascular assembly instead. In a study of Pazgal et al., VEGF-C, VEGF-D, and VEGFR3 were expressed in both lymphoma cells and endothelial cells of the blood and lymphatic vasculature. They reported a significant correlation of the VEGF-C expression and the presence of blood vessels. VEGF-D expression correlated with the patient International Prognostic Index (IPI) Score and the patients’ overall survival (14). These results may indicate that apart from its role as primary signaling pathway for lymphatic vessels, the VEGF-C–VEGFR3 axis also has implications on angiogenic processes of LN blood vessels. A study in breast cancer demonstrated that VEGFR3 is significantly upregulated in the endothelium of new blood vessels. The results also suggested that VEGF-C secreted by the intraductal carcinoma cells acts predominantly as an angiogenic growth factor for blood vessels, although other immune or stromal cells might be involved in this paracrine signaling network as well (290). An experimental study using a *Myc*-driven aggressive lymphoma mouse model, which resembles important aspects of aggressive B-NHL, supported this hypothesis, showing that the MVD expansion was triggered by lymphoma-provided VEGF-C, in a synergistic activity with LTα_1_β_2_ (42).

Representing a high-grade and angiogenesis-associated lymphoma type, multiple clinical trials with anti-angiogenic agents for the treatment of DLBCL have been conducted. Most of the treatment approaches using single agent angiogenesis inhibitors failed to prove a beneficial effect. However, combinatorial treatment strategies such as R2-CHOP (lenalidomide, R-CHOP) (291, 292), brought encouraging results. Such oberservations emphasize that anti-angiogenesis therapies might not be effective when applied alone, even in highly vascularized lymphoma, but are valuable components in combination with other drugs.

**Burkitt’s lymphoma** (BL) represents around half of all malignant non-Hodgkin lymphoma in children and around 2% in adults (293). The BL pathogenicity is usually associated with the infection of B cells with the Epstein-Barr virus (EBV). EBV gene products induce BL cell-derived soluble factors that result in inhibition of neo-vascularization and eventually tumor necrosis and regression (294). However, in *in vivo* experiments, EBV-positive cells induced massive recruitment of leukocytes at the tumor border and the development of granulation tissue with large numbers of blood and lymphatic vessels (295). Surprisingly, aggressive BL displayed the highest MVD in comparison to intermediate DLBCL and indolent B-NHL (42, 262, 287). In support of this observation, BL showed increased vascularization relative to benign lymphadenopathies and can produce several angiogenic factors, although it is not yet known whether this is due to *Myc* gene overexpression or the EBV transformation (296–298). BL were found to be closely associated with VEGF-producing CD68^+^VEGFR1^+^ myeloid cells located around the neo-vasculature. The newly formed blood vessels were identified by the absence of pericyte coverage as result of the rapid vessel growth (299, 300). Genetic depletion of this subpopulation of CD68+VEGFR1+ myeloid cells was sufficient to inhibit angiogenesis in experimental lymphoma (301). To our knowledge, to date there are no clinical data or published treatment strategies of BL that target angiogenesis specifically.

**Classical Hodgkin Lymphoma** (cHL) is characterized by mono-nucleated Hodgkin and multi-nucleated Reed-Sternberg (HRS) cells, which comprises tumors with mixed cellularity, nodular sclerosis and lymphocyte-rich or lymphocyte-depleted subtypes. Different from other lymphoma, HRS cells are the minority of cells within the affected LN. Most of the cells in cHL tumors are cells of the TME, indicating a prominent role of benign immune cells and the LN stroma (302). A crucial role of angiogenesis and increased MVD have been reported for cHL and correlate with a poor prognosis (303). Similar to observations in highly vascularized LNs in an aggressive B-NHL mouse model (42) and in immunohistochemically characterized B-NHL patient specimen (277), in cHL HIF-1α was only moderately expressed (304), suggesting that angiogenesis in cHL is not hypoxia-driven and may utilize other angiogenic pathways instead. In childhood cHL, HRS cells express VEGF, MMP-2 and MMP-9. However, the expression of these factors did not correlate with the MVD and neovascularization level (305, 306). On the other hand, VEGF-D, a ligand for VEGFR3 and usually associated with lymphangiogenesis, is expressed in HRS cells at high abundance and correlated with high numbers of microvessels (307). Moreover, in vitro HRS cell-derived TGF-β, FGF-2, and VEGF supported HUVEC tubulogenesis (308, 309). Secretion of Ltα by HRS cells activated endothelial cells, which enhances adhesion molecule expression and consequently, recruitment of T cells. This mechanism amplifies the inflammatory milieu in the cHL TME through conditioning of the blood vasculature (310).

Commonly attributed to the occurrence of angiogenic M2 macrophages, TAMs are linked to poor outcome in HL. Interestingly, lack of macrophages, but also high numbers of macrophages is associated with a poorer disease-free survival and overall survival, whereas intermediate numbers are associated with a better outcome. This macrophage paradox suggests that a lack of TAMs is beneficial for HL growth, while TAMs have an inhibitory effect with increasing numbers (311). The inhibitory effect seems to be displaced by an adverse effect of TAM-induced angiogenesis, supposedly predominated by CD163^+^ M2-like TAMs (312). High numbers of CD163^+^ TAMs correlate with elevated VEGF-A levels and an increased MVD, indicating that CD163 is an independent prognostic marker in cHL (313). Interestingly, although the particular signaling pathways within TAMs remain elusive, pre-clinical experiments with PI3K-Akt pathway inhibition suggested a connection to macrophage M2-polarization (314, 315), which could be a promising anti-angiogenic intervention clue by prevention of pro-angiogenic activity of M2-like TAMs.

## Anti-Angiogenic Therapies in Combination With Chemotherapies

Cancer therapy earlier than the 1970s was solely focused on targeting the actual cancer cells. Judah Folkman’s discovery that tumor growth is angiogenesis-dependent led to a profound paradigm shift in cancer therapy (316, 317). Sprouting angiogenesis plays an essential role in tumor growth, invasion, progression, and metastasis, targeting this process is a promising strategy to inhibit growth and spread of solid tumors. Clinical trials and treatment strategies of anti-angiogenesis therapy in B-NHL were recently reviewed (217). Angiogenesis inhibitors are classified into direct and indirect agents. Direct inhibitors target vascular ECs and include endostatin, arrestin, and tumstatin. Indirect angiogenesis inhibitors target tumor cells or cells of the TME to prevent the expression of pro-angiogenic factors or block their activity (318). The anti-VEGF monoclonal antibody Bevacizumab was the first anti-angiogenesis drug approved by the FDA for the treatment of metastatic colon, ovarian, renal, non-squamous cell lung cancer, and glioblastoma multiforme. Unfortunately, clinical significance was only reached in glioblastoma multiforme treatment (319, 320), a result that could not be confirmed in other studies (321). In contrast to Bevacizumab, treatment with tyrosine kinase inhibitors (e.g., Sorafenib) that interfere with the signal transmission of VEGFRs resulted in remarkable effects throughout several cancer entities. Combination of tyrosine kinase inhibitors and conventional chemotherapy have not been beneficial (322). Conventional chemotherapy can cause direct cytotoxicity of endothelial cells, but this effect is non-selective and only observed upon the maximal tolerated dose (MTD). Insufficient tumor and vascular bed destruction can effectuate a strong hypoxic condition, which results in release of chemoattractant CXCL12. Accordingly, MTD chemotherapy potentially increases systemic CXCL12, which recruits bone marrow–derived EPCs. These cells can cause recurring angiogenesis in mouse models of solid tumors (323, 324). Therefore, an anti-angiogenesis therapy that is complementary to chemo- or immunotherapy is aimed at restricting pro-angiogenic bystander effects of the tumor treatment. In addition, instead of aiming for a complete vascular eradication, the paradigm in anti-angiogenic therapies shifted to vascular normalization (325, 326).

Rituximab has become an essential part of first-line treatment of several B cell lymphoma entities, foremost of DLBCL. However, ongoing research aims to improve the therapeutic efficiency and the reduction of the relapse rate of drug-resistant lymphoma cells. Tumor anti-angiogenesis therapy approaches are one branch of such research, in which Bevacizumab and Endostatin were the most promising representatives for lymphoma treatment (327, 328). VEGF-A has a crucial role in promoting vessel growth, but is also considered to be an immunosuppressive factor that modulates the migration and function of several immune cells, e.g., DCs and mast cells. The potential pharmaceutical targeting of the VEGF/VEGFR axis to modulate anti-tumor immunity has been reviewed recently (329).

**Figure 3:**
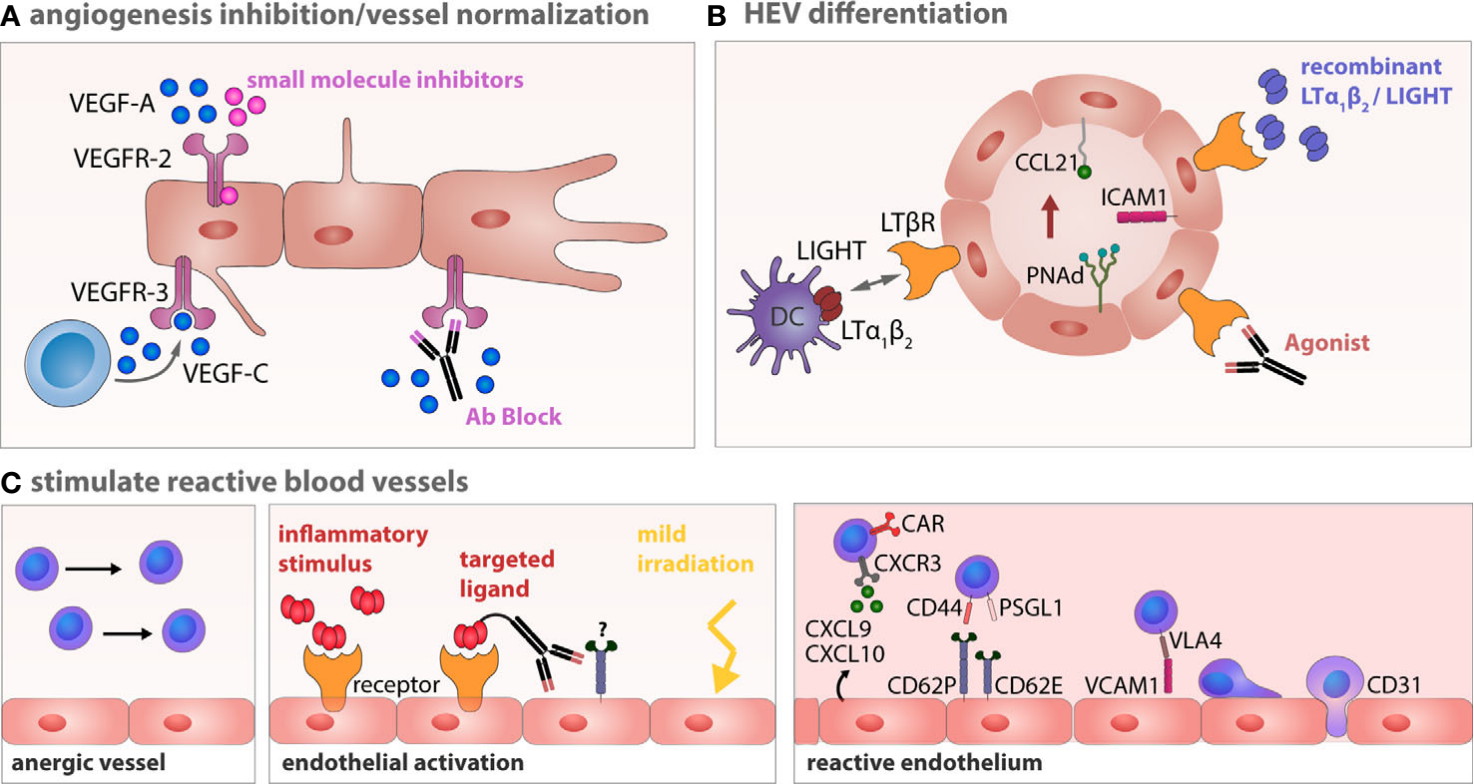
Therapeutic strategies to induce vessel normalization and revert endothelial anergy in B-NHL. **(A)** Anti-angiogenesis therapy tageted at VEGFR-2 or VEGFR-3 can restore a normalized vessel network. **(B)** Targeting of the LTβR with LTα_1_β_2_ and LIGHT expressing DCs, agonistic antibodies or recombinant factors potentially circumvents impaired lymphocyte homing by establishing or stabilizing HEV integrity within the lymphoma TME (338). **(C)** Vessel anergy can be changed by a targeted conversion of the endothelium toward a reactive endothelium using inflammatory cytokines, which might be site directed to avoid unintended systemic effects. Normalization of aberrant vessels and activation of the endothelium can also be achieved by locally applied low-dose gamma irradiation (339). Reactive endothelium within LNs is a prerequisite for an effective infiltration of effector T cells during cellular immunotherapy.

An important challenge of anti-angiogenic therapy in solid tumors as well as in lymphoma is the identification of the particular angioactive receptors throughout different tumor entities and individual patients. The inhibition of intracellular signaling hubs is a strategy to overcome the targeting of distinct angiogenic tyrosine-kinase receptors. Class I PI3Ks are involved in the signal transduction of many pro-angiogenic signals and control cell growth, survival, motility, and metabolism (330). PI3Kδ inhibition in lymphoma potentially also interferes with tonic signaling in tumor cells, e.g., via the BCR signaling pathway (331), or breaks the Treg-mediated immune tolerance (332). Interestingly, PI3K activity is essential for macrophage M2 polarization (333) and therefore, a potential target to hamper M2-like angiogenic macrophages. Inhibition of PI3K signaling represents a valuable therapeutic strategy to target different indolent B cell lymphoma entities, among them FL, CLL, SLL, and more recently, they showed promise in T cell lymphomas as well (334, 335). The combinatorial treatment of the first generation PI3K inhibitor idelalisib with rituximab or bendamustine revealed favorable response rates in FL patients (334), but serious adverse effects due to bacterial and viral infections were observed. Additionally, immune-mediated and hematologic adverse events occurred. Beyond that first generation PI3K inhibitor, newer PI3K inhibitors such as copanlisib and duvelisib were introduced for patients with relapsed and progressive FL, CLL, SLL, respectively. These inhibitors differ in their preference for PI3K isoforms which are expressed differentially in various tissues (336). Despite relevant side effects of PI3K inhibitors, they have been judged clinically manageable and thus, prompted an FDA approval for relapsed and refractory indolent B-NHL (335, 337). Published reports on anti-angiogenic therapies in B-NHL allow the conclusion that the complex mechanisms of angiogenesis in lymphoma are incompletely understood and require further pre-clinical and translational research to develop reliable and effective anti-angiogenic treatment strategies. Moreover, new anti-angiogenic treatment regimens need to be validated regarding an actual reduction of tumor growth, since sole targeting of angiogenic factors often fail to cause substantial tumor regression (**Figure 3**) (340).

## Outlook

Vascular remodeling and angiogenesis have been increasingly recognized as crucial factors in the pathophysiology of B-NHLs. We here present an integrated concept that includes angiogenic processes of the LN TME beyond the proliferation and survival of endothelial cells stimulated by the VEGF-VEGFR axis. In human, the angiogenic properties of LN resident and recruited immune cells are still insufficiently understood. Studies to address such functional states are limited because tissues from appropriate human patients are rarely available. Notably, most of the human data available so far are observations on clinically progressed and even terminal stage lymphoma LNs. Flow cytometry analysis of blood samples is usually limited to a few entities (e.g., FL, CLL, and MCL), common markers and cannot readily be correlated with pathohistological observations due to the lack of tissue specimen. Availability of LN tissue from progressed disease stages is often limited to scarce material from fine-needle biopsies. As a useful surrogate, mouse models of reactive LNs and LNs with lymphoma growth demonstrated that the angiogenic processes are regulated by a timely complex interplay of immune, tumor, and stromal cells (42).

In the future, modern methods like single-cell RNA sequencing alone or with spatial resolution, or single-cell analysis in combination with proteomics will help to resolve the complexity of participating cells and their heterogenous differentiation status. This technique requires much less input material for a high resolution analysis at the genome, protein, or epigenome level (341–343). Even patient-derived specimen from fine needle biopsies seem amenable to such analysis, allowing then a kinetic description of LN remodeling in the course of diagnosis, treatment response, and eventually relapse. Single-cell RNA sequencing will further enable the discrimination of different endothelial cell subtypes and their differentiation traits (344). The compartment of BECs is comprised of several functionally distinguishable subpopulations that further differentiate during angiogenesis. To date, we know little about the role, differentiation conditions and distribution of these subpopulations in LNs. Such transcriptional observations need to be correlated with the topology of the single cells and cell networks within the complex LN (345). Modern imaging methods, e.g., light sheet microscopy and intravital 2-photon microscopy enable the complex spatial integration and the investigation of dynamic processes *in situ*, which have long been restricted to snapshot observations. Very recently, a new generation of flow cytometry devices became available that, based on a spectrum wide detection of fluorophores, allow a simultaneous detection of a multi-fold higher number of cell markers. The possibility to determine extended marker panels with small sample sizes will not only improve basic knowledge in the pre-clinical context, but will also provide innovative approaches for clinical diagnostics (346, 347).

Improving insight into angiogenesis is also of considerable relevance for the emerging immunotherapies using chimeric antigen receptor (CAR)- and T cell receptor (TCR)-transgenic T cells and NK cells. It is reasonable to suggest that tumor blood vessels have a leading role in granting effector T cell access to the LN and the tumor niche formed therein. For example, solid tumors condition an endothelial activation status that can be considered immunologically “silent” (348). However, reactivation of such vessel-lining endothelial layers is a prerequisite for the adhesion and transmigration cascade of naive and therapeutic T cell populations. We envision that this endothelial tuning is not only applicable to solid tumors, but also to LN-localized lymphatic neoplasm. Except for cHL, immune checkpoint blockade (ICB) targeting PD-1 or CTLA-4 has not shown relevant benefit in other B cell neoplasm. Because ICB efficacy depends on the presence of a repertoire of antigen-specific T cells, a rational sequence of immunotherapeutic interference in B-NHL would start with a vessel induction toward a more activated or even inflammatory state. It seems not even necessary to overactivate local endothelial cells, as shown by the application of a modified TNFα cytokine that upregulates adhesion molecules, but then even eradicates solid tumors through rapid destruction of the tumor neovasculature (349). Enhanced adhesion, e.g., involving ICAM-1 and VCAM-1 up-regulation, may be sufficient to allow T cells to get access to the primary lymphoma site in the deep parenchyma. Finally, in a time window to be defined, application of ICB might then unleash the activity of effector T cells that already invaded the tumor site.

Collectively, efforts to target tumor cells only or single lymphoma-promoting cellular stromal elements in the TME are unlikely to confer long lasting remissions. For example, although anti-CD19 CAR T-cell therapies have proven remarkable efficacy in B cell malignancies, they become ineffective due to CD19 antigen loss or downregulation (350, 351). Other contributing factors to substantial rates of treatment failure might be the nodal immunosuppressive microenvironments in B-NHL (9, 352).What is needed is an integrative concept that blocks vicious feedback cycles in lymphoma. We suggest that combinatorial targeting of aberrantly polarized myeloid cell populations, blood endothelial activation, angiogenesis, and effector T cell dysfunction is a rational stepwise strategy (**Figure 3**). Advanced CAR T cell technologies try to integrate a few of these demands, for example by deleting the functionality of PD-1 (353, 354) and secretion of immune-stimulatory cytokines such as IL-12, IL-21, or IL-18 (355–357). We envision that the vasculature is important for control of lymphoma relapse. In this process, mutual stimulation of residual tumor cells, mesenchymal and hematopoietic stromal cells, and endothelial cells might favor neo-angiogenesis and eventually, re-shaping a growth supporting niche for lymphoma B cells.

## Author Contributions

LM: conceived the general idea, wrote the manuscript, and created the figures and tables. AR: conceived the general idea, co-wrote the manuscript, and edited figures and tables. UH: provided expert opinion/knowledge input and edited manuscript, figures, and tables. All authors contributed to the article and approved the submitted version.

## Funding

This work was funded by the Wilhelm Sander-Stiftung (grant number 213.100.02), and by the Deutsche Krebshilfe (grant number 107749) awarded to AR and UH.

## Conflict of Interest

The authors declare that the research was conducted in the absence of any commercial or financial relationships that could be construed as a potential conflict of interest.
